# Supplementary material for: Reliable infarction of the middle cerebral artery territory in C57BL/6 mice using pterygopalatine artery ligation and filament optimization – The PURE-MCAo model
Source: J Cereb Blood Flow Metab. 2024 Oct 7;45(5):871–84. doi: 10.1177/0271678X241281841 (PMC11563556; doi:10.1177/0271678X241281841)
Supplement: sj-pdf-1-jcb-10.1177_0271678X241281841 - Supplemental material for Reliable infarction of the middle cerebral artery territory in C57BL/6 mice using pterygopalatine artery ligation and filament optimization – The PURE-MCAo model [file sj-pdf-1-jcb-10.1177_0271678X241281841.pdf]

Supplementary materials

Reliable infarction of the middle cerebral artery territory in C57BL/6 mice using pterygopalatine artery ligation and filament optimization – the PURE-MCAo model

Sodai Yoshimura[1,2C], Maximilian Dorok[1], Uta Mamrak[1], Antonia Wehn[1, 3], Eva Krestel[1], Igor Khalin[1,4C*], Nikolaus Plesnila[1, 5 C*]

[1]Institute for Stroke and Dementia Research (ISD), LMU University Hospital, Ludwig-Maximilians-University Munich (LMU), Germany; [2]Nihon University School of Medicine, Department of Neurosurgery, Tokyo, Japan; [3]LMU University Hospital, Department of Neurosurgery, Munich, Germany; [4]Normandie University, UNICAEN, INSERM UMR-S U1237, Physiopathology and Imaging of Neurological Disorders (PhIND), GIP Cyceron, Institute Blood and Brain @ Caen-Normandie (BB@C), Caen, France; [5]Munich Cluster for Systems Neurology (Synergy), Munich, Germany

* Authors contributed equally to this work

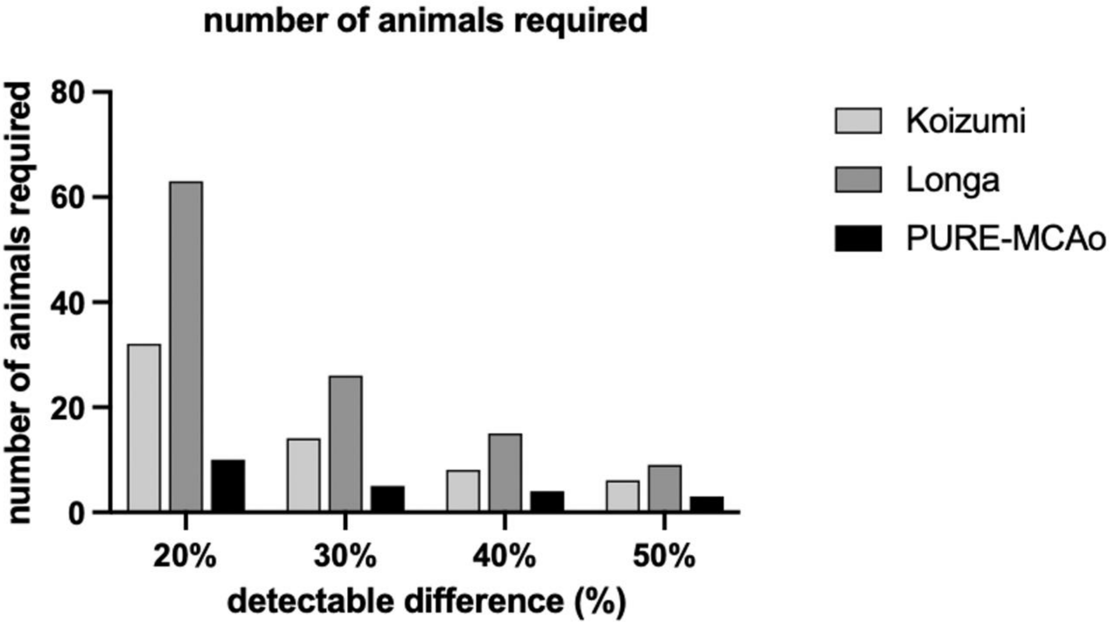

Figure S1. Comparison of animal numbers required in experiments across methods.

A graph shows the number of animals required to achieve each detectable difference (20%, 30%, 40%, and 50%) in infarct volume among the Koizumi method, Longa method, and PURE-MCAo method. Sample size calculations were conducted using the G*Power software, with settings of $\alpha = 0.05$ and power = 0.8.

METHODS

Anesthesia

Anesthesia was initiated with 5% isoflurane and maintained with 2% isoflurane in 30% oxygen and 70% nitrogen. Body temperature was maintained at 36-37°C using a feedback-controlled heating pad (Heater Control Module, FHC, Bowdoinham, ME, USA).

Neurological Severity Score (NSS)

Neurological function was assessed in all mice with the NSS 24 hours after reperfusion. We modified the Bederson score[1] as described below to allow for video-based assessment, facilitating scoring by third parties.

0: No neurological deficit

1: Slight difference in strength between the limbs on the side contralateral to the lesion, with asymmetrical gait during walking.

2: Inability to walk straight due to contralateral paralysis caused by the lesion, resulting in a circling movement.

3: Unable to move from the spot, rotating in place.

4: Severe contralateral paralysis caused by the lesion, unable to maintain posture.

When the degree of neurological symptoms was determined to be positioned between each score, respective scores of 0.5, 1.5, 2.5, 3.5 were assigned.

Statistical Analysis

Statistical analyses were conducted using GraphPad Prism (version 9.4.1, GraphPad software Inc, USA). For comparisons between two independent groups, the Student's t-test was applied when the data followed a normal distribution, while the Mann-Whitney U test was used for data not following a normal distribution. For comparisons between three or more groups, a one-way Analysis of Variance (ANOVA) was performed for data following a normal distribution, whereas the Kruskal-Wallis H test was used for data not following a normal distribution. As post-hoc multiple comparisons, Tukey's test or Dunn's test was performed.

1. Bederson, J.B., et al., *Rat middle cerebral artery occlusion: evaluation of the model and development of a neurologic examination.* Stroke, 1986. **17**(3): p. 472-6.
